# The impact of primary healthcare reform on equity of utilization of services in the province of Quebec: a 2003–2010 follow-up

**DOI:** 10.1186/s12939-015-0243-2

**Published:** 2015-12-14

**Authors:** Marie-Jo Ouimet, Raynald Pineault, Alexandre Prud’homme, Sylvie Provost, Michel Fournier, Jean-Frédéric Levesque

**Affiliations:** Direction de la santé publique du CIUSSS du Centre-Sud-de-l’Île-de-Montréal, 1301 Sherbrooke est, Montréal, Québec H2L 1M3 Canada; Centre for Primary Health Care and Equity, University of New South Wales, Chatswood, New South Wales Australia; Bureau of health information, Level 11, Sage Building, 67 Albert Avenue, Chatswood, New South Wales 2067 Australia

**Keywords:** Equity, Primary care, Primary healthcare, Utilization of services, Health disparities, Healthcare reform, Primary healthcare organization, Family practice

## Abstract

**Introduction:**

In 2003, the Quebec government made important changes in its primary healthcare (PHC) system. This reform included the creation of new models of PHC, Family Medicine Groups (e.g. multidisciplinary health teams with extended opening hours and enrolment of patients) and Network Clinics (clinics providing access to investigation and specialist services). Considering that equity is one of the guiding principles of the Quebec health system, our objectives are to assess the impact of the PHC reform on equity by examining the association between socio-economic status (SES) and utilization of healthcare services between 2003 and 2010; and to determine how the organizational model of PHC facilities impacts utilization of services according to SES.

**Methods:**

We held population surveys in 2005 (*n* = 9206) and 2010 (*n* = 9180) in the two most populated regions of Quebec province, relating to utilization and experience of care during the preceding two years, as well as organizational surveys of all PHC facilities. We performed multiple logistical regression analyses comparing levels of SES for different utilization variables, controlling for morbidity and perceived health; we repeated the analyses, this time including type of PHC facility (older vs newer models).

**Results:**

Compared with the lowest SES, highest SES is associated with less emergency room visits (OR 0.80) and higher likelihood of at least one visit to a PHC facility (OR 2.17), but lower likelihood of frequent visits to PHC (OR 0.69), and higher affiliation to a family doctor (OR 2.04). Differences remained stable between the 2005 and 2010 samples except for likelihood of visit to PHC source which deteriorated for the lowest SES. Greater improvement in affiliation to family doctor was seen for the lowest SES in older models of PHC organizations, but a deterioration was seen for that same group in newer models.

**Conclusions:**

Differences favoring the rich in affiliation to family doctor and likelihood of visit to PHC facility likely represent inequities in access to PHC which remained stable or deteriorated after the reform. New models of PHC organizations do not appear to have improved equity. We believe that an equity-focused approach is needed in order to address persisting inequities.

**Electronic supplementary material:**

The online version of this article (doi:10.1186/s12939-015-0243-2) contains supplementary material, which is available to authorized users.

## Résumé

**Introduction:**

En 2003, le gouvernement du Québec a instauré une réforme des services de santé de première ligne (SPL) qui s’est traduite principalement par la création des groupes de médecine de famille (équipes de soins multidisciplinaires avec heures d’ouverture étendues et inscription de patients) et des cliniques-réseau (permettant un meilleur accès aux plateaux techniques et aux spécialistes). L’équité étant l’un des principes centraux du système de santé québécois, nous nous proposons d’évaluer l’impact de la réforme des SPL sur l’équité en examinant l’association entre le statut socio-économique (SSE) et l’utilisation des services de santé entre 2003 et 2010 ainsi que l’impact des modèles d’organisation de services de SPL sur cette association.

**Méthodologie:**

Nous avons mené une enquête téléphonique en 2005 (*n* = 9206) et 2010 (*n* = 9180) dans les deux régions les plus peuplées du Québec, portant sur l’utilisation des services de santé durant les deux années précédentes, ainsi qu’une enquête organisationnelle de toutes les organisations de SPL. Nous avons réalisé des analyses de régression logistique multivariée en comparant les niveaux de SSE selon diverses variables d’utilisation, en contrôlant pour la morbidité et la santé perçue; nous avons répété les analyses en incluant le type d’organisation de SPL (anciens vs nouveaux modèles).

**Résultats:**

Comparativement au plus faible SSE, le SSE le plus élevé est associé à une probabilité moindre de visite à l’urgence (RC 0,80) et une probabilité plus élevée d’au moins une visite en SPL (RC 2,17), mais une probabilité moindre de visites fréquentes en SPL (RC 0,69 ) et une affiliation plus élevée à un médecin de famille (RC 2,04). Ces différences demeurent stables entre 2005 et 2010 sauf pour la probabilité d’au moins une visite en SPL qui s’est détériorée pour le plus faible SSE. L’affiliation au médecin de famille a davantage augmenté pour le SSE le plus faible dans les anciens modèles, mais elle a connu une plus grande détérioration pour ce même groupe dans les nouveaux modèles.

**Conclusions:**

Les différences observées en faveur des riches représentent vraisemblablement des iniquités d’accès à la première ligne qui sont demeurées stables ou se sont détériorées après la réforme. Les nouveaux modèles de SPL ne semblent pas avoir amélioré l’équité. Une approche centrée sur l’équité apparaît nécessaire pour réduire les iniquités persistantes.

## Introduction

Access to healthcare and socio-economic status (SES) are well-known determinants of health [1]. The link between these two determinants of health has found growing interest in recent years [2, 3]. Although universal healthcare systems aim to provide healthcare services according to need rather than ability to pay, in OECD countries, and in Canada in particular, it has been shown that access varies greatly and that utilization of services is not merely distributed according to need [2, 4–6]. Important factors involved are levels of income as well as education, social support and region of residence [7].

Inequities refer to differences that are judged unjust or unfair [8–10]. Although use of specialist services almost systematically shows a pro-rich distribution [11, 12], most industrialised countries with a universal health system have improved equity in the use of primary care services: in general, people with greater needs receive more primary care services [13]. But some degree of inequity still remains, such as pro-rich inequity in the number of visits to general practitioners (GP) for several European countries [11], fewer visits to the GP by people of lower SES in several OECD countries including Canada [2, 12], and pro-rich inequity in the probability of a GP visit in most Canadian provinces [6]. Some authors have even suggested that in recent years, inequities in utilization of primary care services may have appeared or increased [11, 12] in several European countries. Even though inequity in utilization is not strictly synonymous with unfairness in accessibility, as utilization is also dependent on individual preferences, need for care, expected benefit of care, as well as ability to seek care and to engage in the process of care [6, 14], looking at utilization as a proxy for accessibility is a widespread practice [7, 15]. Also, not all inequalities or inequities in health can be solved by improved accessibility for those more in need of services. Action on other social determinants of health often plays a key role [16].

As mentioned above [2, 6], evidence suggests that there are persisting inequities in healthcare use in Canada, which is provided by provincial governments and is covered universally for hospital and physician services in all provinces. Equity is one of the guiding principles of the Quebec Health and social services system, which aims to ensure equitable access to quality care and services for all citizens [17]. This translates into policies aiming to facilitate access to healthcare in order to match utilization with health needs, especially for vulnerable populations. Differences exist between provinces in coverage for medication, as well as paramedical services such as psychotherapy, dentistry or physiotherapy; the province of Quebec, historically known to be one of Canada’s most social-democratic provinces, offers the most thorough, though still incomplete, coverage for these.

Traditionally, Quebec PHC organizations have been divided into privately-owned clinics such as solo and group practices, where few other professionals are involved and opening hours vary greatly. Local community services centers (LCSC) are public clinics that were created in the early 1970s to provide health and social services. They are the most involved for socially vulnerable populations. LCSCs include a large proportion of nurses, social workers and psychologists, but their populational impact is very small as few patients are followed. Finally, family medicine units (FMU) are teaching units which share most characteristics of LCSCs.

In 2003 the Quebec government launched a reform, introducing Health and Social Services Centers (HSSC), local structures responsible for the coordination of all healthcare services in one specific geographical area and entrusted to form Local Health and Social Services Networks (LHSSN). The reform included the creation of new models of primary care that resulted from the transformation of the above-mentioned older models (LCSC, solo practice, group practice). The first model is the family medicine group (FMG) [18, 19]. An FMG consists of 6 to 10 physicians who work consistently with nurses, and often other professionals (dieticians, psychologists and/or social workers), to provide services to enrolled patients on a non-geographical basis (10,000 to 20,000 patients per FMG). It offers increased accessibility through extended opening hours and participation in a regional on-call system (Table 1). In addition, under the initiative of Montreal Regional Health Agency, a complementary model of PHC organizations was implemented, the Network clinic (NC). A NC is more specifically aimed to improve accessibility through walk-in visits and provides better access to technical support, such as X-rays and laboratory tests, and to specialists. The distinction between FMG and NC is often difficult to establish, as many clinics have acquired both status, and thus benefit from two sources of funding, provincial and regional.

**Table 1:** Percentage of PHC organizations with specific organizational characteristics by type, 2005 and 2010

Organizational characteristics		FMG-NC^a^ (*N* = 16)		FMG^b^ (*N* = 86)		NC^c^ (*N* = 17)		LCSC^d,e^ (*N* = 40)		Group practice^e^ (*N* = 208)		Solo practice^e^ (*N* = 172)	
		2005 (%)	2010 (%)	2005 (%)	2010 (%)	2005 (%)	2010 (%)	2005 (%)	2010 (%)	2005 (%)	2010 (%)	2005 (%)	2010 (%)
Presence of nurses	Yes	77.8	100	66.3	83.7	70.6	94.1	97.5	97.5	23.1	27.9	13.4	18.6
Presence of specialists and/or other health professionals in the same building	Yes	88.9	100	90.7	79.0	94.1	88.2	85.0	90.0	86.6	76.4	58.2	44.1
Information technologies used in the practice	At least one	83.3	94.4	81.4	90.7	70.6	100	92.5	97.5	55.8	66.3	33.1	43.6
Collaboration with other PHC practices	Yes	27.8	66.7	61.6	80.2	76.5	76.5	32.5	42.5	42.3	22.6	41.3	24.4
Collaboration with hospitals	Yes	50.0	94.4	61.6	74.4	64.7	76.5	57.5	72.5	44.2	37.5	40.1	27.3
Opened on evenings (after 6 PM) and week-ends	Yes	88.9	88.9	81.4	80.2	100	94.1	80.0	75.0	69.2	48.6	49.4	36.0
Predominant type of visits in the practice	Walk-in visits^f^	33.3	16.7	14.0	2.3	64.7	41.2	7.5	7.5	29.3	19.2	10.5	8.7
	By-appointment visits^g^	22.2	11.1	62.8	57.0	5.9	5.9	77.5	77.5	51.9	58.7	81.4	83.1
	Mixed^h^	44.4	72.2	23.3	40.7	29.4	52.9	15.0	15.0	18.8	22.1	8.1	8.1

^a^Family Medicine Group and Network Clinic (double status)

^b^Family Medicine Group only

^c^Network Clinic only

^d^Local Community Services Centre

^e^Without FMG or NC status

^f^>50 % of all visits are walk-in visits

^g^≥ 75 % of all visits are by-appointment visits

^h^26 to 50 % of all visits are walk-in visits

Through the creation of these new structures, the Quebec reform aimed to improve access and continuity in healthcare, as well as improve coordination of services [20]; this has been the object of formal evaluations [21–24]. There is growing literature linking access to healthcare with the models of organization of PHC [18, 19]. Some studies link organizations such as FMGs to better accessibility of services [23]. However, although the concern for equity has been explicitly voiced by governmental bodies [20], no formal evaluation of the equity implications of the Quebec reform has been undertaken to this day. One would expect a reduction in inequalities due to the longstanding equity tradition of Quebec, but the literature on structural reforms and their impact on equity, though scarce, suggests that the opposite may occur in some contexts [25].

This paper is part of a project that aimed to follow the evolution of PHC models and its impact on patients’ experience of care [26]. The main goal of the project was to identify models of PHC which are most adapted to the needs of the population, in order to inform clinicians and policymakers on the effects of the reform. The objective of this specific study is to examine the association between SES and utilization of healthcare services and its evolution between 2003 and 2010 in Quebec, and secondarily, to explore how organizational models of PHC (newer vs older) might impact utilization of services according to SES.

## Methods

### Study design

This study follows a longitudinal strategy with a natural experiment design without a control group, comparing two repeated independent samples of the population, in 2005 and 2010, and repeating a survey of all PHC organizations during the same time period.

### Data source

The project consisted in two population-based telephone surveys of randomly-selected adults from the two most populous regions in the province of Quebec, Montréal and Montérégie. Using the random-digit dialing method, approximately 400 respondents were recruited in each of 23 local networks, for a total of 9206 respondents in the 2005 sample (response rate of 64 %). The survey was repeated in 2010 with 9180 respondents (response rate of 56 %). Special attention was given to optimize response rates by the firm involved in the survey: many calls (maximum 140, mean 8.4) were made for each phone number; an alternative web-based questionnaire was offered to respondents who had refused the phone interview [27, 28].

The first survey provided a reference point for further comparison, as most elements of the reform were only partially implemented in 2005 [29–32], and questions referred to the two years preceding the survey. The survey allowed to assess the evolution of population-level experience of care up to seven years into the reform. The questionnaire covered demographic characteristics, income, education, morbidity, perceived health, as well as several questions relating to healthcare utilization and experience of care during the previous two years (see Additional file 1). Respondents who did not speak French or English were excluded, as well as those with significant disability interfering with the survey process.

Health services utilization was established by asking participants if they were affiliated with a family physician; if they had visited a family physician in the past two years, and if so, how often; if they had visited an emergency room in the past two years; and if they had been hospitalized in the past two years. Those who had visited a family physician were asked to identify their main source of PHC. Even though our focus was on PHC, emergency room (ER) use and hospitalization were assessed because they are considered sensitive to access of PHC: better access to PHC is associated with lower use of ER and hospitalization rates [33].

In recent years, wealth has been thought to uncover a qualitatively different pattern of inequality that may be concealed by traditional measures of economic status such as income [34]. Moreover, it has been suggested to include other measures of economic status to income level to form a more accurate and balanced picture when using survey methodology [35, 36]. Therefore, rather than using income as our only economic status indicator, we constructed a composite index combining annual crude income adjusted to size of household (divided into quartiles), perception of economic status (range: poor to well-off) and number of assets (car, house, savings), using a formative approach [37–41]. We refer to this index as SES even though education, which is often part of such indicators, is used as a separate variable in our models; using economic status alone as an indicator of SES is a commonly used approach [42]. Our index has since been widely used by our research team [43]. Each item is intended to represent a distinct conceptual dimension of SES, which is confirmed by the fact that items are not highly correlated with each other. Values of SES range from zero to ten. The score was divided according to groups that were evident in the distribution into four categories as follows: 0 to 3.6: very low SES; 4.6 to 6.4: low; 7.3 to 8.2: high; 9.1 to 10: very high.

A composite index of morbidity was computed using self-reported numbers of cardiovascular risk factors (hypertension, diabetes, dyslipidemia) and numbers of chronic diseases (asthma/chronic obstructive pulmonary disease (COPD)/other respiratory illness, coronary artery disease (CAD)/heart failure/other cardiac illness, arthritis, stroke). Again, a formative approach was used. In most comparable surveys, morbidity is measured by computing the number of chronic diseases cumulated by an individual. Our measure is similar though it also includes a number of cardiovascular risk factors. This index was then divided into four sub-categories. Again, this index has been widely used by the research team [26, 31, 32, 43] and has been shown to predict hospitalization and ER use.

In addition to our morbidity index, we included perceived health as a separate health status variable, as it is often used as a complement to diagnosed illness in studies on healthcare utilization [44, 45].

All population-level data were weighted by attributing subjects the inverse probability of selection, in order to account for unequal sampling probabilities resulting from both local area sampling and intra-household selection. In addition, a post-stratification weighting comparing with census data was applied for age and sex distribution.

The project also included two surveys of PHC organizations which were conducted in 2005 and 2010 in the two same regions (see Additional file 2). A questionnaire was mailed to key informants in all PHC organizations of both Montréal and Montérégie. Questions related to vision, structure, resources and practices of the various sources of PHC [26]. The types of PHC sources existing in administrative databases as well as these organizational surveys can be divided as follows: family medicine groups (FMG), network clinics (NC), clinics having both FMG and NC status (FMG-NC), local community services centres (LCSC without FMG/NC status), family medicine teaching units (FMU without FMG/NC status), group clinics (involving more than one physician--- not FMG/NC) and solo clinics (involving only one physician).

### Data analysis

**Fig. 1:**
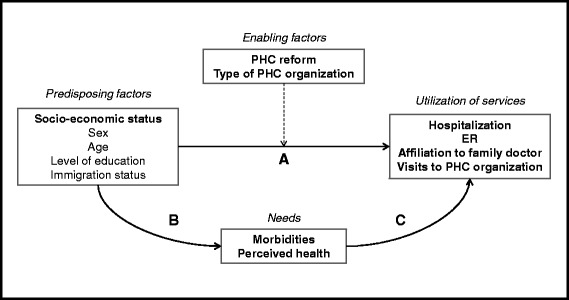
Conceptual framework

We first examined the association of SES with different outcomes representing utilization of health services, while controlling for socio-demographic and health status variables (detailed below), for both survey years (2005 and 2010). We tested all relevant variables according to our conceptual framework [46] (Fig. 1), within the limitations of available data. We selected variables that were statistically significantly associated with at least one outcome in bivariate analysis and performed multiple logistical analyses using STATA version 10.0 with all respondents (*n* = 18386) using the variables below. Analyses were made for 2005 and 2010 jointly, and interaction terms between year and SES were created in order to detect differential responses between economic groups. Need variables (morbidity and perceived health) were modelled as mediator variables in our final models, as shown in our conceptual framework [45, 46].

#### 1st objective: variables

*Outcome variables (utilization)*: “at least one hospitalization in past two years” (yes-no), “at least one visit to ER in past two years” (yes-no), “affiliation to a family doctor” (yes-no), “at least one visit to PHC source in past two years” (yes-no).

*Control variables (predisposing factors)*: age, sex, level of education,^1^ immigration status (born in Canada, immigrated to Canada less than 10 years ago, immigrated to Canada 10 years ago or more).

*Mediator variables (needs)*: morbidity level (no cardiovascular risk factor^2^ or chronic disease,^3^ at least one cardiovascular risk factor, one chronic disease with/without risk factor, at least two chronic diseases with/without risk factor), perceived health (poor/average, good, very good, excellent).

*Predictors (predisposing/enabling factors)*: year (2005 or 2010), SES (very low, low, high, very high).

*Interaction variables*: year × SES.

In order to address our second objective, i.e. how the pre- vs post-reform organizational model of PHC might impact utilization of services according to SES, we repeated the above analyses but this time we introduced the type of PHC source in our model. All six previously mentioned categories of PHC sources were separated into two broad types: older models (LCSC/FMU, group clinic, solo clinic) and newer models (FMG, NC, blended model (FMG-NC)). Interaction terms between year and SES were maintained, and new interaction terms between year, type of PHC and SES were added in order to detect differential responses between these three variables.

#### 2nd objective: variables

*Outcome variables (utilization)*: “at least one hospitalization in past two years” (yes-no), “at least one visit to ER in past two years” (yes-no), “affiliation to a family doctor” (yes-no).

*Control variables (predisposing factors)*: age, sex, level of education, immigration status (born in Canada, immigrated to Canada less than 10 years ago, immigrated to Canada 10 years ago or more).

*Mediator variables (needs)*: morbidity level (no cardiovascular risk factor^2^ or chronic disease^3^, at least one cardiovascular risk factor, one chronic disease with/without risk factor, at least two chronic diseases with/without risk factor), perceived health (poor/average, good, very good, excellent).

*Predictors (predisposing/enabling factors)*: year (2005 or 2010), SES (very low, low, high, very high), type of PHC (older model, newer model).

*Interaction terms:* year × SES, year × type of PHC, SES × type of PHC, SES × year × type of PHC.

For these analyses, only respondents who had declared a PHC source were included (*n* = 12951). Outcome variables therefore did not include “at least one visit to PHC source” as this was the criterion to define users of a PHC source. The analyses were performed using the 2010 type and comparing results for the same clinics in time; for example, a clinic that had become a NC in 2010 was included in the NC group in 2005.

Finally, we analyzed the association of SES with frequency of utilization of PHC using the following outcome variable for users of PHC services only (*n* = 12951): “at least six visits to PHC source during the past two years” (yes-no). There is no consensus in the literature about the definition of frequent users of PHC [47]. Some authors choose a number of visits [48], while others prefer to establish a threshold in the distribution in order to allow better comparison between settings [49]. Limitations related to our questionnaire (see Additional file 1) and distribution of our data pointed towards a cut-off point of 6 visits. Additional analyses using different thresholds (available from the authors upon request) led to the same conclusion. Again, all other variables remained the same, and analyses were performed with and without the variable “ type of PHC ” in our model.

For all analyses, odds ratios (OR) with their 95 % confidence intervals (CI) were computed. Where interaction terms were shown significant, adjusted probabilities were calculated and schematized. As mentioned above, all analyses were weighted to account for unequal probabilities of sampling which arise from the stratified two-stage sampling as well as for age and sex distribution.

### Ethics approval

Our study was performed in accordance with the principles of the Helsinki Declaration. The Research Ethics Committee of the “Agence de la santé et des services sociaux de Montréal” gave approval for the study.

## Results

Demographic information on the 2005 and 2010 samples is shown in Table 2. Based on census data, the 2005 sample was representative of the general population with regard to all variables except level of education. The 2010 sample differed significantly from the 2005 sample, as more respondents were in the higher education categories. Proportions of respondents in the low and high SES also differed slightly between both years, as well as the age composition, which is slightly older in 2010. Table 3 shows the distribution of respondents according to the utilization variables for all levels of SES. Also included is the distribution of respondents according to variable “has a usual source of PHC” which is not included in subsequent models, for reference purposes only.

**Table 2:** Characteristics of respondents (2005: *n* = 9206; 2010: *n* = 9180) (weighted samples)

Subject characteristics		2005 (%)	2010 (%)	p (Chi-2)
Sex	M	48.5	48.7	.783
Age	18-29	20.6	19.7	.031
	30-44	28.4	27.2	
	45-64	33.5	34.6	
	65+	17.5	18.5	
Level of education	<Secondary	15.8	12.5	<.001
	Secondary	32.5	30.1	
	College	24.2	20.8	
	University	27.5	36.7	
SES^a^	Very low	11.4	10.0	<.001
	Low	32.8	30.9	
	High	29.9	32.6	
	Very high	25.9	26.5	
Immigrant status	Born in Canada	80.5	79.5	.261
	Has immigrated <10 years	6.3	6.6	
	Has immigrated ≥10 years	13.2	13.9	
Morbidity	None	49.5	48.5	.096
	≥1 risk factor^b^	16.8	18.0	
	1 chr. disease^c^	25.6	25.9	
	≥2 chr. diseases	8.2	7.6	
Perceived Health	Poor or average	16.3	14.6	.001
	Good	28.4	29.7	
	Very good	34.7	34.0	
	Excellent	20.5	21.8	

^a^Socio-economic status

^b^Without chronic disease. Cardiovascular risk factors: hypertension, diabetes, dyslipidemia

^c^With/without risk factor. Chronic diseases: asthma/COPD/other respiratory illness, CAD/heart failure/other cardiac illness, arthritis, stroke

**Table 3:** Distribution of respondents according to utilization of services, by SES, 2005 (*n* = 9206) and 2010 (*n* = 9180) (weighted samples)

Utilization variable		Very low		Low		High		Very high		Total	
		2005 (%)	2010 (%)	2005 (%)	2010 (%)	2005 (%)	2010 (%)	2005 (%)	2010 (%)	2005 (%)	2010 (%)
Has a usual source of PHC		63.2	65.4	67.7	76.8	74.7	82.7	75.1	81.1	71.2	78.8
≥1 hospitalization past 2 years		21.6	24.9	15.9	19.0	13.7	18.3	12.5	13.7	15.0	18.0
≥1 ER visit past 2 years		37.9	40.1	31.9	38.5	30.8	32.5	27.1	31.8	31.0	34.9
≥1 visit to PHC past 2 years		78.5	73.3	79.4	82.7	85.9	86.1	88.0	85.4	83.5	83.6
≥6 visits to PHC past 2 years^a^		30.7	27.0	26.5	22.6	20.5	17.1	15.9	12.9	22.2	18.4
PHC type^a^	FMG-NC	9.7	12.8	9.8	10.4	9.7	10.7	10.8	12.4	10.0	11.2
	FMG	21.4	21.5	26.4	26.2	27.3	27.6	25.5	26.4	25.9	26.3
	NC	11.8	11.1	9.6	8.9	7.7	8.9	7.4	8.1	8.6	8.9
	Total newer models	42.8	45.4	45.8	45.4	44.7	47.2	43.7	46.8	44.6	46.4
	LCSC	9.9	9.7	7.6	6.7	6.0	3.5	5.1	4.7	6.6	5.3
	Group practice	40.9	36.4	39.2	37.9	41.0	40.0	43.8	40.6	41.2	39.2
	Solo practice	6.3	8.6	7.5	10.0	8.3	9.2	7.4	8.0	7.6	9.1
	Total older models	57.2	54.6	54.2	54.6	55.3	52.8	56.3	53.2	55.4	53.6
Affiliation to family doctor		58.4	65.0	66.1	70.9	71.1	78.4	72.0	76.8	68.2	74.3

^a^Among individuals who have a usual source of PHC (n^2005^ = 6198; n^2010^ = 6753)

For all logistical regression analyses results, though results relating to control variables may be of interest, they will not be discussed as our focus is on SES.

Results of the analyses corresponding to our first objective, i.e. the association between SES and utilization of healthcare services, and its evolution between the 2005 and 2010 surveys, are shown in Table 4. For all levels of SES, whether tested together or separately, there is no significant difference between years 2005 and 2010 for likelihood of hospitalization.

**Table 4:** Factors associated with utilization of services among all respondents in past two years (*n* = 18386), 2005 and 2010 samples combined (logistical regression)

Variables in model		Hospitalization			ER			Affiliation to family doctor			≥1 visit to PHC past 2 years		
		OR	95 % CI		OR	95 % CI		OR	95 % CI		OR	95 % CI	
Year (ref.: 2005)	2010	1.257	.981	1.610	1.141	.923	1.409	.955	.747	1.222	**.689**	.525	.903
SES	Low	.**806**	.652	.995	**.824**	.695	.978	**1.460**	1.212	1.758	1.118	.898	1.391
(ref.: Very low)	High	.862	.688	1.079	.859	.717	1.029	**1.881**	1.548	2.286	**1.812**	1.432	2.293
	Very high	.918	.718	1.173	**.796**	.656	.966	**2.035**	1.650	2.511	**2.168**	1.667	2.819
Interaction year × SES	Low × 2010	1.044	.772	1.411	1.216	.945	1.565	1.064	.797	1.422	**1.799**	1.301	2.489
(ref.: Very low x 2010)	High × 2010	1.163	.857	1.578	1.003	.778	1.292	1.227	.918	1.640	**1.408**	1.009	1.963
	Very high × 2010	.931	.671	1.293	1.132	.867	1.477	1.167	.863	1.577	1.190	.837	1.694
Sex (ref.: Male)	Female	**1.387**	1.250	1.540	.955	.882	1.035	**1.837**	1.684	2.004	2.319	2.086	2.578
Age	30-44	.935	.795	1.099	**.828**	.732	.937	**1.458**	1.288	1.651	**1.197**	1.029	1.392
(ref.: 18–29)	45-64	**.652**	.552	.771	**.548**	.483	.622	**2.591**	2.277	2.949	**1.214**	1.038	1.421
	65 or over	.895	.742	1.081	**.445**	.382	.519	**5.137**	4.286	6.158	1.168	.953	1.432
Level of education	Secondary	**.814**	.698	.948	1.003	.881	1.142	1.029	.882	1.200	1.181	.997	1.401
(ref.: <Secondary)	College	**.771**	.649	.916	.981	.849	1.133	.924	.784	1.091	**1.466**	1.208	1.778
	University	**.729**	.616	.864	.892	.774	1.027	.873	.741	1.028	**1.446**	1.200	1.743
Immigrant status	Has immigrated <10 years	.893	.704	1.133	.718	.600	.860	**.524**	.435	.632	**.646**	.528	.789
(ref.: Born in Canada)	Has immigrated ≥10 years	1.013	.866	1.184	1.064	.945	1.198	.977	.855	1.115	.970	.824	1.142
Morbidity	≥1 risk factor	1.159	.991	1.355	1.094	.974	1.230	**2.398**	2.100	2.738	**2.929**	2.465	3.480
(ref.: None)	1 chr. disease	**1.540**	1.347	1.761	**1.476**	1.332	1.634	**1.722**	1.537	1.928	**2.313**	1.992	2.687
	≥2 chr. diseases	**2.863**	2.399	3.419	**2.429**	2.084	2.832	**2.657**	2.165	3.261	**3.429**	2.605	4.514
Perceived health	Good	**.579**	.507	.663	**.655**	.583	.735	1.127	.980	1.297	**.748**	.626	.895
(ref.: Poor/Average)	Very good	**.398**	.344	.461	**.514**	.456	.580	**1.191**	1.034	1.372	**.698**	.583	.835
	Excellent	**.383**	.322	.455	**.425**	.369	.489	1.133	.970	1.324	**.533**	.441	.644

Note: Statistically significant results (*p* < 0.05) are in bold

Slightly different results apply to ER use (Table 4). Globally, there is no difference in likelihood of an ER visit between both sample years, but when taken separately, the low and very high SES are less likely to visit the ER than other levels of SES (low SES 0.82, CI 0.70-0.98; very high SES OR 0.80, CI 0.66-0.97). There is no evidence that the observed differences may have changed between the 2005 and 2010 samples, as interaction terms between year and SES are not significant.

Likelihood of affiliation to a family doctor is unchanged between the 2005 and 2010 samples globally. However, likelihood of affiliation increases concurrently with SES (low SES OR 1.46, CI 1.21-1.76; high SES OR 1.88, CI 1.56-2.29; very high SES OR 2.03, CI 1.65-2.51). Interactions between year and SES are again not significant. Thus, when we translate these results into adjusted probabilities, we find that in the 2005 sample, 59 % of the very low SES people were affiliated to a family doctor, compared with 58 % in 2010 (non significant). In contrast, 72 % of the very high SES people were affiliated to a family physician in 2005, compared with 74 % in 2010 (data available upon request).

**Fig. 2:**
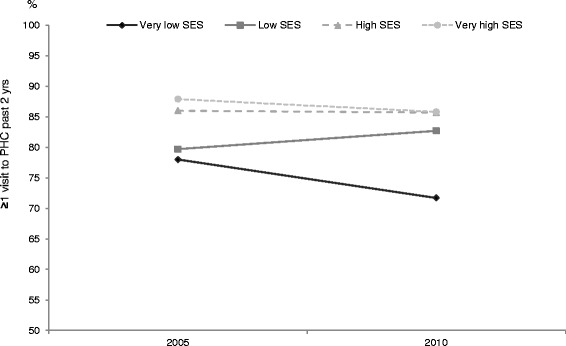
Probability of at least one visit to PHC source in past two years according to socio-economic status

Respondents of all SES levels combined are less likely to declare having visited a PHC source in 2010 than in 2005 (OR 0.69, CI 0.52-0.90). However, individuals in the high and very high SES are more likely to have visited a PHC source (high SES OR 1.81, CI1.43-2.29; very high SES OR 2.17, CI 1.67-2.81) than the very low SES. This time, interactions between year and SES are significant for the low and high SES, suggesting that observed differences between levels of SES have changed in 2010 compared with 2005 (Fig. 2). In fact, the gap has decreased between the three higher SES groups, but it has widened with the very low SES.

**Fig. 3:**
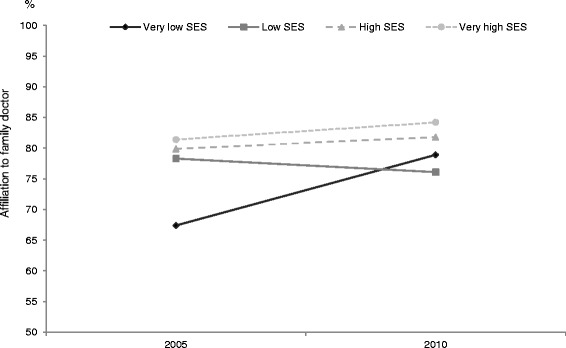
Probability of affiliation to a family doctor according to socio-economic status (old models of PHC)

**Fig. 4:**
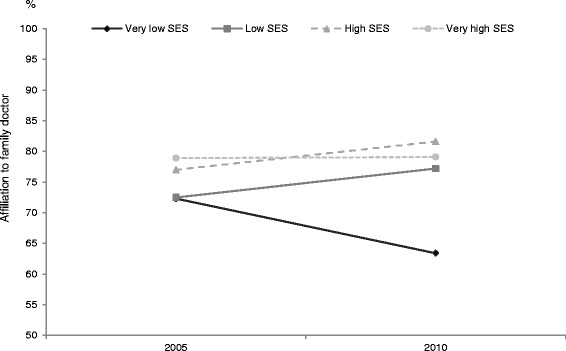
Probability of affiliation to a family doctor according to socio-economic status (new models of PHC)

Analyses which included type of PHC source were restricted to respondents who had declared at least one visit to a PHC source, since respondents who had not visited PHC could not be linked with a given source. Results for likelihood of hospitalization, ER use and frequency of use of PHC were comparable to results of analyses performed previously. However, there appears to be a differential relationship between year and SES when we examine adjusted probability of affiliation to a family doctor for old vs new types of PHC. This relationship is schematized in Fig. 3 (old models of PHC) and Fig. 4 (new models PHC). Affiliation to a family doctor appears to have improved between the 2005 and 2010 samples for the very low SES group more than for other groups within the old models; conversely, affiliation has deteriorated for the very low SES group in the new models whereas it has improved slightly for the other SES groups.

Finally, we examined the likelihood of having six or more visits to the PHC source among those respondents who had at least one visit to PHC (Table 5). We found no evidence that the likelihood of having six or more visits to PHC had changed between the 2005 and 2010 samples for all levels of SES combined. Likelihood of high frequency of use (six or more visits) decreased concurrently with SES but was only significant for the very high group (OR 0.69, CI 0.53-0.90). Observed differences between SES groups remained stable between the 2005 and 2010 samples as interaction between year and SES was not significant. This time, including type of PHC source did not suggest a differential effect of type of PHC model on equity of utilization (data available upon request).

**Table 5:** Factors associated with high utilization (≥6 visits) of PHC services among users in past two years (*n* = 12951), 2005 and 2010 samples combined (logistical regression)

Variables in model		≥6 visits to PHC past 2 years		
		OR	95 % CI	
Year (ref.: 2005)	2010	.789	.595	1.045
SES	Low	.923	.727	1.173
(ref.: Very low)	High	.824	.642	1.057
	Very high	**.690**	.527	.903
Interaction year × SES	Low x 2010	1.042	.743	1.461
(ref.: Very low x2010)	High x2010	1.005	.715	1.412
	Very high x2010	1.094	.756	1.581
Sex (ref.: Male)	Female	**1.365**	1.218	1.530
Age	30-44	.920	.755	1.121
(ref.: 18–29)	45-64	.846	.700	1.024
	65 or over	1.031	.834	1.276
Level of education	Secondary	.948	.805	1.116
(ref.: <Secondary)	College	**.749**	.624	.900
	University	**.752**	.626	.903
Immigrant status	Has immigrated < 10 years	.771	.566	1.052
(ref.: Born in Canada)	Has immigrated ≥10 years	.968	.820	1.143
Morbidity	≥1 risk factor	**1.807**	1.542	2.116
(ref.: None)	1 chr. Disease	**1.773**	1.532	2.051
	≥2 chr. diseases	**3.086**	2.539	3.752
Perceived health	Good	**.706**	.609	.817
(ref.: Bad/Average)	Very good	**.502**	.428	.588
	Excellent	**.337**	.273	.415

Note: Statistically significant results (*p* < 0.05) are in bold

## Discussion

Our results show differences in utilization of healthcare services between socio-economic groups. Higher SES people are less likely to visit the ER and be high-frequency users of PHC. These two utilization variables being closely related to one another, it is not surprising that results point in the same direction. Conversely, our results show that lower SES individuals are less likely to be affiliated to a family doctor, and also less likely to report at least one visit to a PHC source. Again, these two last variables are closely linked. All observed differences are stable between 2005 and 2010 except for likelihood of at least one visit to PHC source. For this last indicator, the situation seems to have improved in 2010 in favor of the low and high SES, but has deteriorated for the very low SES.

Our results suggest differences when we include type of PHC source: there appear to be observable differences in equity between older and newer models of PHC concerning affiliation to a family doctor. Inequities appear to have improved in the old models, and to have deteriorated in the new models.

Whether some of the observed differences in favor of the lowest SES actually translate into equity depends on an accurate measurement of need factors. In our study we included morbidity, but our measure was only partial, computing risk factors and chronic diseases but not their severity; perceived health may be a more accurate measure of severity of illness but again it is incomplete. Nevertheless, the higher likelihood of utilization by the disadvantaged could mean that equity exists for use of ER services and, to a lesser extent, frequency of use of PHC once PHC is accessed, considering that the poor have greater needs for services.

The differences in favor of the rich, observed in affiliation to a family doctor and likelihood of at least one visit to PHC source, suggest that there are inequities in accessing family physicians and PHC source, and that some of these inequities have worsened despite the PHC reform. The fact that in 2010, after controlling for other variables, 58 % of people in the lowest SES category were affiliated to family doctors compared to 74 % in the highest SES category is disturbing from a policy perspective. Even more disturbing is the fact that while for the high and very high SES, 86-87 % of individuals visited a PHC source during both study periods, this proportion has fallen from 78 to 72 % for the very low SES during the same period.

The results showing that affiliation to family physicians has remained stable for all SES groups in 2010, while likelihood of at least one visit to PHC has decreased (OR 0.69), suggest that affiliation to a family doctor does not necessarily equate with access to PHC. The fact that more nurses were involved in PHC in 2010 could act as a confounding factor that our data could not control for.

More importantly, results suggesting that affiliation to family doctors improved for the very low SES group in old models of PHC, but deteriorated in the new models, seem to imply that the reform failed to improve equity of PHC delivery on that important aspect. Official registration with a family physician was first implemented in new models, which may mean that the registration process itself could lead to increased inequities in attributing patients to physicians.

Our analysis supports previous Canadian and international studies which suggested that removing financial barriers to healthcare is insufficient to ensure equity in utilization of services [2, 4–6, 50–54]. However, since the literature is relatively scarce about the equity implications of health reforms in countries with universal healthcare systems [25, 55] and methodologies differ significantly, it is difficult to make comparisons on that important aspect of our study.

The differences we have observed between SES levels for ER use cannot be explained only by higher disease prevalence since our analysis controls for a number of risk factors and chronic diseases; therefore we hypothesize that lower SES could be associated with delayed access, leading to health status deterioration and increased severity of disease (as discussed above, we did not have an accurate measure of severity of disease), which in turn would lead to higher use of ER services and, to a lesser extent, higher frequency of use of PHC source, as was also suggested by other authors [51, 56–58]. It is likely that individuals of higher SES are able to benefit more effectively from the healthcare system.

This would also explain why the disadvantage observed in likelihood of visit to PHC for the lowest SES has deteriorated; the complexity associated with all PHC structures, old and new, after the reform, may have played a role in this respect. A recent study performed in Quebec supports this hypothesis: complexity of the healthcare system was mentioned as one of the main barriers to seeking and benefitting from care for deprived individuals [59]. New financial barriers implemented during the study period such as administrative fees, although they are still not used consistently in the Quebec healthcare system, may have further widened the gap in utilization of PHC between the rich and the poor; unfortunately our data does not allow us to confirm this hypothesis.

Poor health literacy [58, 59] could certainly make navigating through the healthcare system more challenging; this is particularly true about registration with a family physician, which is often perceived as a complex process. New structures may have become even more complex than the older ones, which could partly explain the differences we have observed between old and new PHC models. Also, the fact that affiliation to a family physician in Quebec is voluntary certainly needs to be questioned further when discussing the equity impacts of this reform. Although there are incentives for registering vulnerable populations with a GP, SES has not been considered a criterion for vulnerability by the Quebec healthcare system administrative rules. A rostering system in favor of the disadvantaged could help to complement other aspects of the reform in improving equity. In fact, the equity of attribution of patients through access registries has recently been questioned [60] and should be thoroughly examined for improvement. Complementary solutions recently suggested by the actual Quebec Ministry of Health, such as increasing the number of patients per family physician and widely adopting advanced access, while promising, need to be carefully planned and implemented with a concern for equity in order to improve rather than increase existing inequities.

Asada [5] suggests that processes involved in use versus frequency of use of services may differ. Frequency depends more on the professional’s decision, often referred to as secondary demand for services, while use or non-use depends more on the individual’s decision, also known as primary demand for care. That could explain why results for both variables do not point in the same direction.

Most importantly, we feel that our results support the conceptual framework for access to healthcare [14] which suggests that equity of access depends on a series of preliminary conditions in order for an individual to obtain and benefit from services: ability to perceive the need for, ability to seek and ability to reach services. In our study, we examined hospitalizations and ER use, which are highly dependent on need; affiliation to family physician and use of PHC services, which depend both on perception of need and ability to seek and reach care; and intensity of use of PHC, which represents ability to reach and to engage in care. These characteristics, all along the continuum of access to healthcare, belong to the demand-side, whereas the Quebec structural reform mostly affected supply-side aspects such as approachability and availability of services, while setting aside important aspects such as acceptability and appropriateness of services for vulnerable populations [59]. This assessment has been shared by outside observers [61] who felt that the reform addressed supply-side issues but that little was made to raise public awareness to the changes it involved, and that therefore there was no true demand-side pressure from the general public. The demand-side focus in Quebec has been on access to ER and not so much on PHC until recently. Furthermore, though the tendency is to generalize access to multidisciplinary teams, patients may not be ready yet for such a change, and not all patients benefit equally from such an approach [62].

### Study limitations

Our study has some limitations. First of all, as discussed previously, our morbidity measure is only partial and does not take into account the severity of disease which is likely to be worse for lower-income individuals. On the other hand, lower-income people, who have a lower rate of consultation for most preventive services [63], may be less aware of their risk factors than the more advantaged population. Despite these limitations, our index has previously shown to be a good predictor for use of services, as mentioned above.

Also, the survey form of the study leads to the possibility of a recall bias: the perception of an individual’s utilization is not as accurate as would be the use of administrative databases, but it is more compatible with a patient-centred perspective. Also, we do not believe that this type of bias should be stronger in one group in particular.

The sample size and relatively good response rates allow us to have confidence in our results. Other surveys of the kind show similar response rates [54]. We do not have information on non-respondents, therefore it is impossible to determine the extent of non-response bias, but this in turn is minimized by the use of weighted samples. Also, although the samples differ in their composition, the fact that we used weighted samples and controlled for major socio-demographic variables minimizes the risk of bias.

The nature of the phenomenon observed led to the natural experiment design of this study. No control group could be used, since the whole population is submitted to changes in the health system. Therefore changes that may have occurred which were not due to the reform could not be controlled for.

**Fig. 5:**
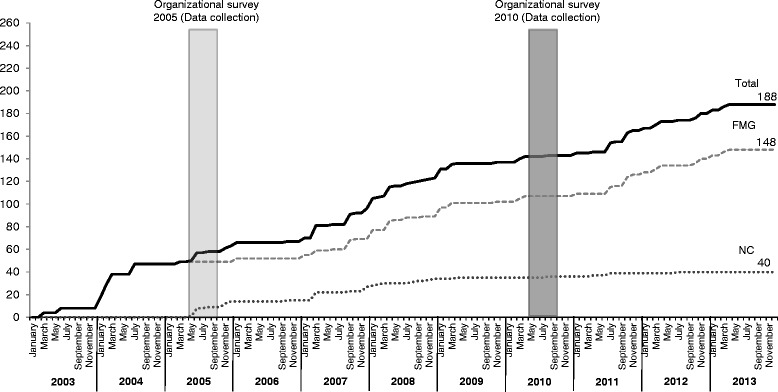
Number of accredited FMG and NC by month and year, Montréal and Montérégie, 2003 to 2013

Our data goes back to 2010. Between 2010 and 2015, many more FMGs and NCs were created; many clinics therefore lost their group status (Fig. 5). However, we have reason to believe that the situation since 2010 is similar, since most changes that were eventually added to the creation of the new PHC models, such as access registries and registration of patients, were implemented before 2010.

## Conclusions

Our study has suggested the presence of pro-rich inequity in affiliation to a family doctor and likelihood of visit to PHC services; some of these inequities appear to have increased between 2003 and 2010 despite the PHC reform. Our study has also suggested that, where affiliation to a family doctor is concerned, the older models of PHC may have become more equitable after the reform, but that inequities may have increased within the newer models. More studies will be needed in order to understand the impact of the organizational model of PHC source on equity, but we feel that a structural reform in itself may not be sufficient to address existing inequities. Demand-side issues should also be addressed by increasing public awareness, thus improving health literacy and the process of care-seeking. As Quebec is entering a new era of reforms and especially of its PHC system once again, lessons can be learned from the previous reform that appears to have failed to improve equity. An equity-focused approach should be central to any future healthcare reform.

## Additional files

Additional file 1:
**Population questionnaire.** (PDF 198 kb) Additional file 2:
**Organization questionnaire.** (PDF 323 kb)

## Abbreviations

CAD: Coronary artery disease
CI: Confidence interval
COPD: Chronic obstructive pulmonary disease
FMG: Family medicine group
FMU: Family medicine teaching unit
GP: General practitioner
HSSC: Health and Social Services Centers
LCSC: Local community services center
LHSSN: Local health and social services network
NC: Network clinic
OECD: Organisation for economic cooperation and development
OR: Odds ratio
PHC: Primary healthcare
SES: Socio-economic status
SES: CIUSSS Centre intégré universitaire de santé et services sociaux

## Footnotes

1 Correlation between education and ES = 0.34.

2 Cardiovascular risk factors: hypertension, diabetes, dyslipidemia.

3 Chronic diseases: asthma/COPD/other respiratory illness, CAD/heart failure/other cardiac illness, arthritis, stroke.
